# An uncommon cause of hemoptysis: aortobronchial fistula

**DOI:** 10.1186/s40248-018-0146-3

**Published:** 2018-09-10

**Authors:** Matteo Fontana, Roberto Tonelli, Filippo Gozzi, Ivana Castaniere, Alessandro Marchioni, Riccardo Fantini, Francesca Coppi, Filippo Natali, Elisabetta Rovatti, Enrico Clini

**Affiliations:** 1 Department of Medical and Surgical Sciences, University of Modena Reggio Emilia, Modena, Italy; 2 Respiratory Diseases Unit and Centre for Rare Lung Diseases, University Hospital of Modena, Modena, Italy; 3 Cardiology Unit, University of Modena, Modena, Italy; 4 Respiratory Diseases Unit, University Hospital S. Orsola Malpighi, Boologna, Italy

**Keywords:** Aortobronchial fistula, Hemoptysis, Aortic surgery

## Abstract

**Background:**

Hemoptysis is a frequent sign of respiratory and non-respiratory diseases. While in most cases the underlying cause is rapidly identified, sometimes the real etiology might be misdiagnosed with dramatic delay in treatment.

**Case presentation:**

A 46-year-old man with hiatal hernia and a history of aortic surgery for aortic coarctation presented with dramatic episodes of hemoptysis and subsequent severe anemia (6,9 g/dl). Digestive and respiratory endoscopy resulted not exhaustive, thus he underwent a contrast-enhanced computed tomography (CT) scan of the chest that showed an aneurysmal dilatation of the descending thoracic aorta with suspected aortobronchial fistula. He underwent cardiac surgery that confirmed the diagnosis and successfully treated the fistula.

**Conclusion:**

We briefly review the literature to raise clinical awareness on this uncommon cause of hemoptysis.

## Background

A 46-year-old man with mild esophageal hiatal hernia and a history of cardiothoracic surgery for aortic coarctation presented with several dramatic episodes of hemoptysis with subsequent severe anemia due to aortobronchial fistula, probably a late consequence of the past aortic intervention. He underwent endovascular aortic intervention with successful management of the fistula. Despite being not very frequent as surgical case, aortobronchial fistula is not so rare as seems and unfortunately it is most of the time a fatal complication. Incidence itself can be underestimated, as the majority of cases are not recognized if a postmortem examination is not performed. With this case report we present the diagnostic work up and treatment of this underhand condition in order to better characterize the spectrum of its presentation and to raise clinical awareness on its dramatic consequences.

## Case presentation

**Fig. 1:**
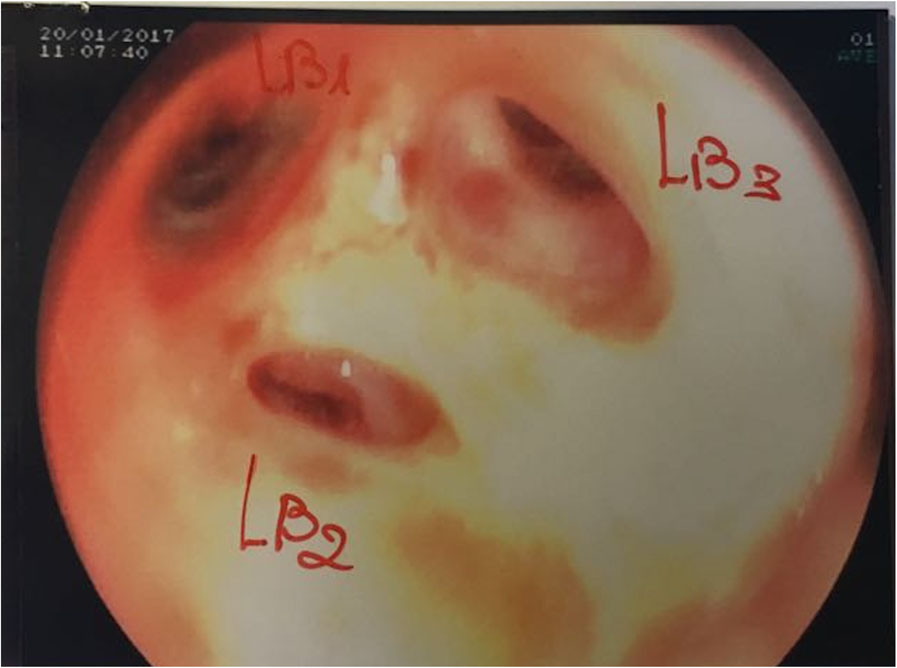
Endoscopic image of the left upper lobe showing residual traces of bleeding

**Fig. 2:**
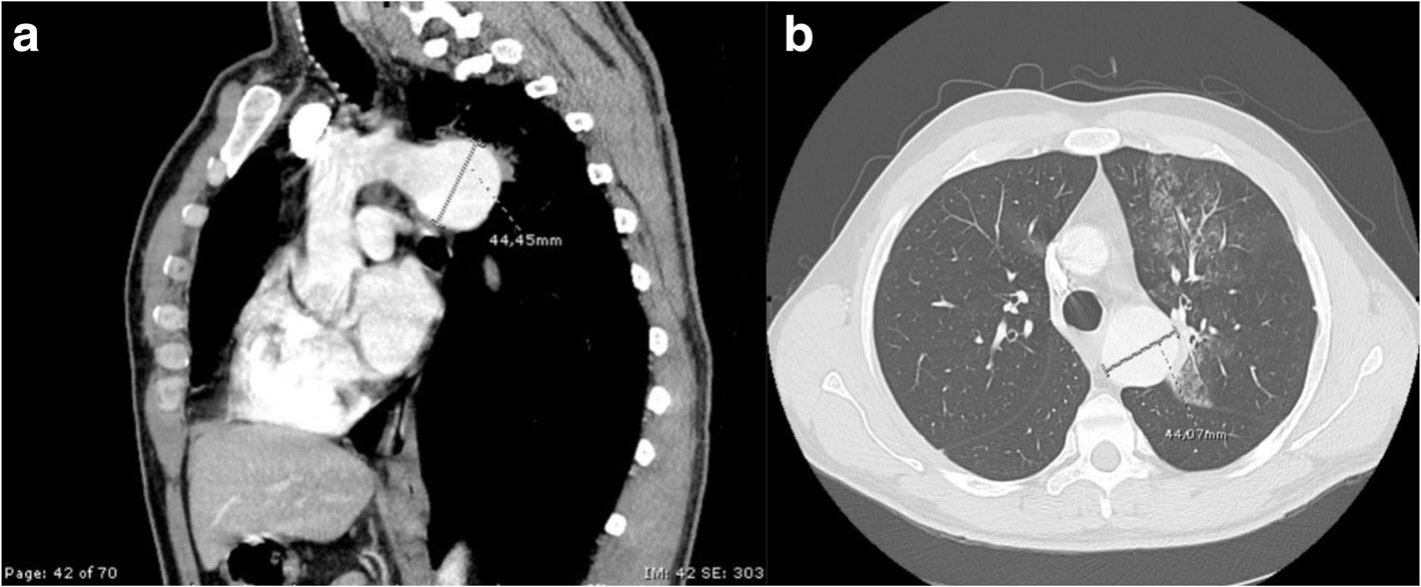
Panel **a** Longitudinal reconstruction of chest CT scan image showing the aneurysmal dilatation of thoracic aorta. Panel **b** Axial reconstruction of chest CT images showing the maximal diameter of the aortic aneurism (44,07 mm) and the ground glass opacities at the left upper lobe

**Fig. 3:**
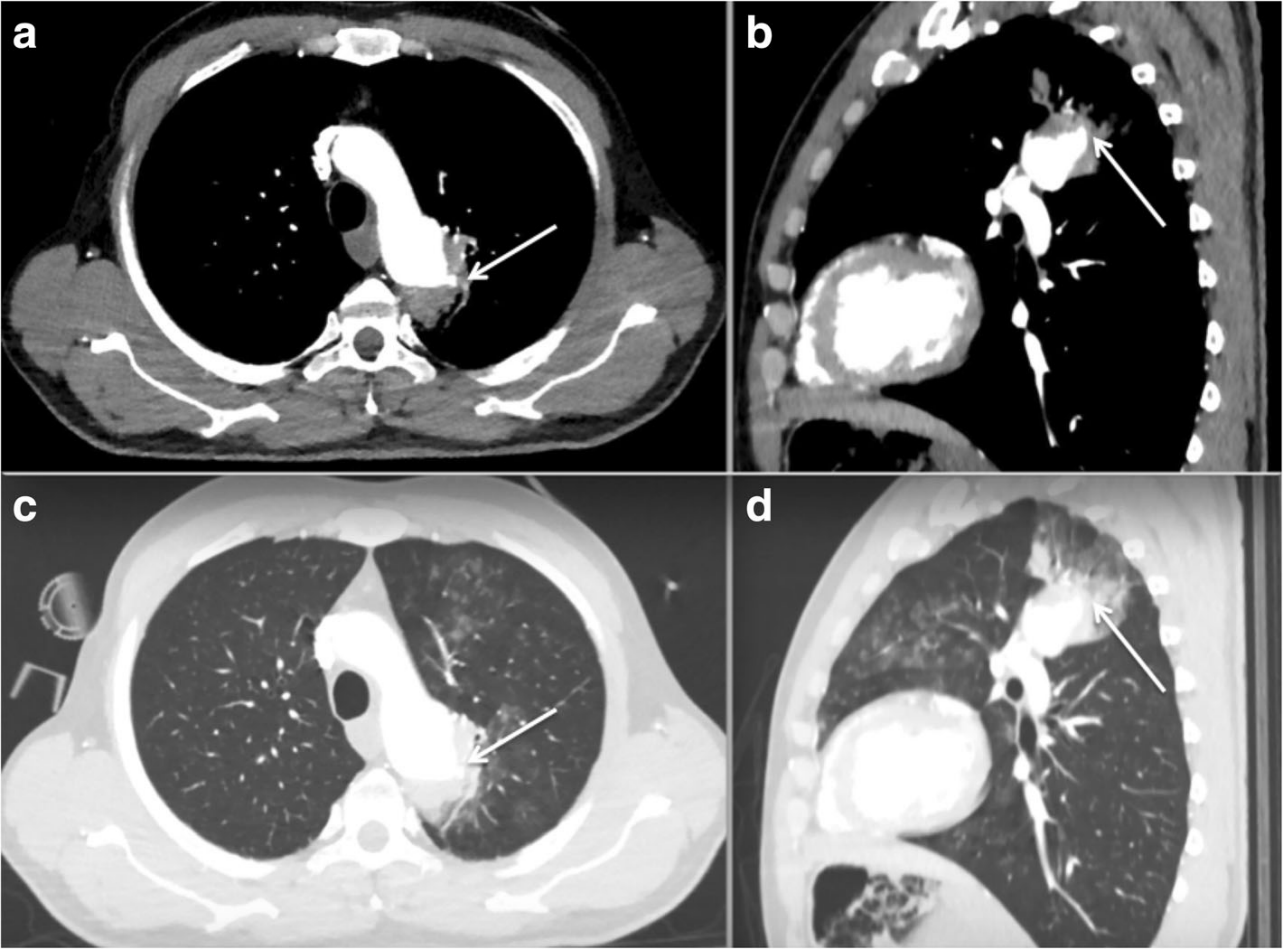
Chest CT scan images lung windows (panels **a** and **b**) and mediastinal windows (panels **c** and **d**) showing aneurysmal dilatation of thoracic aorta with upper posterior leak (arrows) and hemorrhagic parenchymal spreading

A 46-year-old man with a recent diagnosis of hiatal hernia was admitted to the Respiratory Diseases Unit of the University Hospital of Modena, Italy for several dramatic episodes of hemoptysis during the previous 30 days, severe anemia (6,9 g/dl) and initial signs of hemodynamic instability (shock index = 1,4). The past medical history revealed that the patient had undergone cardiac surgery for aortic coarctation at the age of 18 without complications neither during the immediate post-operative course nor in the following 20 years follow up period. He was referred to the Respiratory Intensive Care Unit of our Department where blood transfusion was immediately started. A chest X-ray was performed but no significant abnormalities were detected. Thus he underwent urgent digestive endoscopy that revealed a grade B esophagitis according to Los Angeles classification [1] without any evidence of recent bleeding. Fiber bronchoscopy was then immediately conducted showing limited traces of blood in the bronchial tract afferent to the left upper lobe while no sings of active bleeding was found (Fig. 1). He eventually underwent a contrast-enhanced CT scan of the chest that showed an aneurysmal dilatation of the descending thoracic aorta (Fig. 2) communicating with the left upper bronchus, whose upper posterior hemorrhagic leak determined initial left upper lobe compression and ground-glass opacities with scissural delimitation (Fig. 3). Given the evidence of a communication between aortic aneurism and lung parenchyma or either the tracheobronchial tree the patient was referred to the Cardiac Intensive Care Unit. Thoracic endovascular aortic repair (TEVAR) was preferred rather than a more invasive open surgical approach due to the persistent hemodynamic instability of the patient. Aortobronchial fistula was thus successfully treated with endovascular stent-graft without complications. The patient survived the intervention with uneventful postoperative course and good recovery in less than 30 days. Strict follow up was then started.

## Discussion and conclusions

Since its first systematic description, aortobronchial fistula remains a rare condition characterized by acute symptomatology such as hemoptysis sustained by massive endobronchial bleeding [2, 3]. It represents a misdiagnosed disease especially in patients with coexistent clinical complaints with underestimated incidence and more than 30% cases diagnosed at autopsy [4]. In more recent years the etiology of this unusual condition has been better characterized [5], being now mostly associated with a history of aortic surgery [6, 7]. Studies have showed that complications may occur even many years after the intervention [8], being lethal sequelae of aortic aneurysmal disease [9, 10]. Fistulas usually involve the left side of the bronchial tree because of the narrow distance between the descending thoracic aorta and the left bronchial hemi system, while on the right side the greater distance between the ascending aorta and bronchial tree make this condition unusual [6, 9]. Nevertheless several case reports describing fistulas from the ascending aorta to the right bronchial tree are reported. Aortic fistulas both into the left and right bronchial tree can follow aortic surgery after unpredictable periods, being often the consequence of pseudo-aneurysms [5]. Once the presence of the fistula has been established a rapid multidisciplinary decision regarding further management should be made considering comorbidity, risk factors and clinical stability. In the past the open surgical approach was the only available with prosthetic graft replacement, patch closure or direct suturing of the aortic side of the fistula [11]. Several complications have been described when patients with aortobronchial fistulas undergo open surgery: stroke, paralysis, respiratory failure, acute renal insufficiency, ischemic cardiac events, acute hemorrhage and secondary graft infection [9, 11]. The reported mortality rates range from 25 to 41% [3, 12]. Thoracic endovascular aortic repair with endovascular stent grafting is a simpler and less invasive approach to exclude the fistulous tract with reduced morbidity and mortality, particularly in high risk and unstable patients [13]. Although less invasive, the technique presents some limitations, mainly due to graft contamination, leakage and migration [11, 13]. Furthermore a variety of combinations of TEVAR with surgical aortic repair have been proposed but further studies are needed to assess the long-term efficacy and safety of these techniques [14].

This clinical report is intended to raise attention on this uncommon but dramatic cause of massive hemoptysis.

## Abbreviations

CT: computed tomography
TEVAR: Thoracic Endovascular Aortic Repair
